# How to Generate Self-Efficacy despite Pain: The Role of Catastrophizing and Avoidance in Women with Fibromyalgia

**DOI:** 10.3390/biomedicines12010047

**Published:** 2023-12-24

**Authors:** Patricia Catalá, Lorena Gutiérrez, Carmen Écija, Cecilia Peñacoba

**Affiliations:** Department of Psychology, Rey Juan Carlos University, Avda. de Atenas s/n, 28922 Alcorcón, Madrid, Spain; patricia.catala@urjc.es (P.C.); lorena.gutierrezh@urjc.es (L.G.); carmen.ecija@urjc.es (C.É.)

**Keywords:** fibromyalgia, pain severity, pain catastrophizing, self-efficacy, pain avoidance, moderated-mediation model

## Abstract

Background and Objective: Fibromyalgia-related pain is influenced by numerous factors, including severity, as well as cognitive profiles based on pain catastrophizing or activity patterns. In this context, self-efficacy is identified as a potential predictor for explaining certain health outcomes. This study aimed to contribute to exploring the role of pain avoidance (as activity pattern) between pain severity and self-efficacy along pain catastrophizing. Methods: Through a cross-sectional study, a total of 264 women with fibromyalgia completed self-report measures of pain severity, pain avoidance, pain catastrophizing, and self-efficacy. The severity of the symptoms, the time elapsed since diagnosis, and the time elapsed since the onsets of symptoms were included as covariates to control. Regression-based moderated-mediation analysis was used to test the conditional effect of pain severity on self-efficacy via pain avoidance at varying levels of pain catastrophizing. Results: Pain avoidance mediated the effect of pain severity on self-efficacy. The indirect effects showed a moderated effect when patients scored high on the pain catastrophizing scale. The model evaluated, where catastrophic pain moderates the indirect effect of pain intensity on self-efficacy through pain avoidance, explained 49% of the variance. Conclusions: Catastrophic beliefs associated with pain as being uncontrollable increase the relationship between pain severity and pain avoidance. In turn, pain avoidance is associated with a low perception of capacity.

## 1. Introduction

Fibromyalgia (FM) is considered a syndrome characterized by the presence of persistent and widespread musculoskeletal pain [1]. However, the treatments used to mitigate the symptomatology of these patients have shown a limited efficacy [2]. In recent years, multicomponent treatment (e.g., combination of pharmacological, psychological, and physical activity treatments) has been found to be the most effective guideline for these patients [3,4]. Previous research indicates that although the effects of psychological and pharmacological treatment can be considered similar, psychological treatment (cognitive-behavioral therapy) maintains positive effects at two-year follow-ups [5,6]. One of the main changes produced by cognitive-behavioral therapy is related to the perception of pain control [7]. However, different authors have pointed out that the improvements obtained are mainly due to the increase in self-efficacy in pain management [8,9]. 

Self-efficacy refers to the set of beliefs about one’s own abilities to cope with adverse situations. In terms related to the fibromyalgia disease, it refers to the ability to cope with increased pain, or other characteristic symptoms such as fatigue, in daily activities [10]. Therefore, self-efficacy seems essential in explaining symptomatology and its effect on various areas of patients’ quality of life [11]. In relation to physical activity, several studies have suggested that self-efficacy is related to lower disability and agreeable emotional states by reducing anxious-depressive symptomatology [8,9,12]. However, some authors seem to indicate the need to consider self-efficacy, together with other cognitive processes (e.g., catastrophizing or fear of feeling pain), as explanatory factors of the low adherence to physical exercise in this population [13,14]. 

Within the context of chronic pain, catastrophizing has been widely discussed in terms of its conceptualization and its assessment [15]. Catastrophizing is defined as cognitive processing based on heightened or exaggerated thoughts about pain. Specifically, pain catastrophizing refers to the tendency to focus excessively on the painful sensation, magnify the damage, and perceive the said sensation as uncontrollable [15]. Thus, catastrophizing has been considered one of the key processes in the intensity of symptomatology in fibromyalgia patients [16,17]. In this regard, it has been found that patients with high levels of pain catastrophizing present greater difficulties in shifting attentional focus from painful cues, as they mistakenly consider that these cues precede more intense pain [18]. This fact has been related to an increased fear of pain-related changes, which often leads to a behavioral pattern characterized by excessive rest being highly detrimental in chronic pain processes [14,19]. 

With regard to the pain catastrophizing assessment, the complexity of the concept and the need to adopt a person-centered approach that includes contextual information and expert judgement have recently been pointed out [15]. Crombez et al. [15] found that the content of self-reported assessment measures of pain catastrophizing is closer to a worrying conception related to pain than to a catastrophizing concept. Pain-related worry has been defined as a chain of thoughts charged with a negative effect on pain, and pain catastrophizing has been defined as the process that exacerbates the adverse effects of worry. In this sense, it is proposed to rename the “pain catastrophizing” measures to “pain-related worrying” [15]. This reflection about the “pain-related worrying” highlights the relevance of beliefs and thoughts regarding the impact of pain in the performance of activities. In this context, given that catastrophizing plays an important role in patients with chronic pain, and fibromyalgia in particular, it would be of interest to analyze its influence on the promotion of self-efficacy as a variable involved in patients’ quality of life. Previous research has explored the role of pain catastrophizing and self-efficacy in FM patients’ physical activity and the perceived subjective impact of the disease [20].

Furthermore, based on previous research [10,21,22], where the impact of pain catastrophization on the perception of pain severity has been demonstrated, it could be hypothesized that pain avoidance could mediate the relationship between pain severity and self-efficacy, taking into account the moderating effect of pain catastrophizing on the relationship between pain severity and pain avoidance.

In this context, the main objective of this study is to explore which variables influence the development of adaptive beliefs about pain management ability, considering the contextual effect of pain catastrophizing. Specifically, we aim to investigate whether pain catastrophizing moderates the indirect effect of pain intensity on self-efficacy through pain avoidance. 

## 2. Materials and Methods

### 2.1. Design

A cross-sectional design was used. This study was conducted according to the ethical guidelines of the Declaration of Helsinki, and the research protocol was reviewed and approved by the Bioethics Committee at Rey Juan Carlos University (Reference PI17/00858).

### 2.2. Participants

According to worldwide statistical data, it is estimated that the prevalence of FM reaches 95% in women while diagnoses in men are only around 5% [1]. For this reason, we selected a convenience sample of women diagnosed with fibromyalgia. All women were part of different associations from various Spanish regions in 2019. Following the sample criteria for modelling analysis [23], a minimum sample size of 200 participants was established. The total study sample was made up of 240 women. The inclusion criteria to participate in the present study were the following: be women, have a diagnosis of fibromyalgia according to the American College of Rheumatology criteria for Fibromyalgia [1,24], be over 18 years of age, provide written consent to participate, and have a prescription of walking but not present physical impairments to carry out any physical activity. The exclusion criteria were as follows: not having the physical and mental capacity to give informed consent and complete the surveys.

### 2.3. Measures

#### 2.3.1. Pain Severity

Pain severity was assessed as the mean score of the pain severity items from the Brief Pain Inventory [25]. This scale is composed of four items: minimum, maximum, and general intensity of pain during the last 7 days and intensity of pain in the present moment. It is evaluated through an 11-point scale, ranging from 0 (no pain) to 10 (worst pain imaginable). This procedure for measuring pain intensity has been widely used in the pain literature [26]. Cronbach’s alpha was 0.86 in this study.

#### 2.3.2. Pain Catastrophizing

The Spanish adaptation of the Pain Catastrophizing Scale was used [27]. The scale consists of 13 statements containing a series of thoughts and feelings that people may experience when they are in pain. The items are divided into the dimensions of magnification, helplessness, and rumination. In order to reduce the number of statistical analyses, it was decided to use the global score in this study. This minimizes the risk of false positive errors. In the present study, the alpha value was 0.88 for total score. Higher scores in the scale represent a higher tendency to catastrophize.

#### 2.3.3. Pain Avoidance

The Activity Patterns Scale (APS) was used to assess pain avoidance [28]. This scale assesses eight types of activity patterns: pain avoidance, activity avoidance, task-contingent persistence, excessive persistence, pain-contingent persistence, as well as three pacing patterns, each of which relates to a single goal (increasing activity levels, conserving energy for valued activities, and reducing pain). Each factor is made up of 3 items and is rated using a 5-point Likert-type scale ranging from 0 (not at all) to 4 (always). For the purpose of this study, we only used the pain avoidance pattern scale because the main objective of the present study focuses on pain management in the motivational context advocated by the new theoretical models of pain [15]. For this study, we found a Cronbach’s alpha of 0.78.

#### 2.3.4. Pain Self-Efficacy

The Self-efficacy Questionnaire in Chronic Pain was used [29]. Only the total score was used, obtained from the sum of the responses to the 22 items that make up the questionnaire. It is answered with a Likert-type response scale from 0 (I look totally incapable) to 10 (I look totally capable). The patient must answer the degree to which they consider themselves capable of performing certain activities or managing their pain, their emotional problems, or other symptoms associated with chronic pain. High scores indicate a high perception of self-efficacy. Cronbach alpha in our sample was 0.89.

#### 2.3.5. Symptom Severity Scale (SSS)

To evaluate the severity of symptoms during the last week, the SS scale was used [1]. This scale is composed of the sum of three symptoms, that is, fatigue, non-renewed awakening, and cognitive symptoms, plus the extent of somatic symptoms in general. The minimum total symptom severity score was 0 and the maximum total score was 12. Cronbach’s alpha in our sample was 0.87.

#### 2.3.6. Demographic and Clinical Characteristics

An ad hoc questionnaire conducted by the research team was used to assess age (years), marital status (married or in a stable relationship, single, and divorced or widowed), educational level (primary, secondary, and higher), and employment status (housewives or workers outside the home). Regarding clinical data, time elapsed since FM diagnosis (years) and time elapsed since onset of symptoms (years) were also recorded.

### 2.4. Procedure

Firstly, to recruit the sample, the research team contacted different FM associations in Spain with the aim of explaining the research project and requesting their collaboration. The associations interested in participating contacted their members (fibromyalgia patients who met the inclusion criteria), and a date was agreed upon for the research team to travel to the association to carry out the assessment, thus avoiding patient travel and ensuring greater participation. At the beginning of the evaluation, participants were informed of the objective, procedure, and methodology of the study by signing the informed consent. All recruited patients agreed to participate freely and could withdraw consent at any time. Then, they were given a questionnaire booklet that took approximately 30 min to complete. Two psychologists, to ensure that there were no missing data, supervised completion of the booklet, clarifying any doubts that may arise.

### 2.5. Statistical Analyses

**Figure 1:**
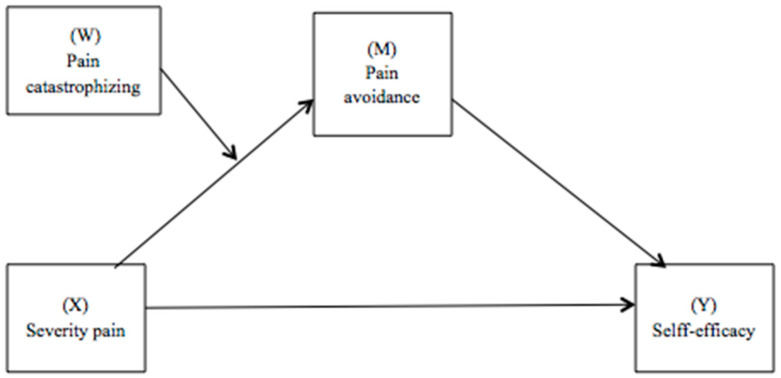
Path diagram illustrating the moderate mediation model.

The IBM SPSS statistics 22.0 software [30] and PROCESS macro v3.3 for SPSS [31] were used for data analysis. Descriptive analyses (e.g., mean, standard deviation, and sample range) were carried out to evaluate the characteristics of the sample and the distribution of the variables under study. The correlations between the main variables were examined using Pearson’s correlation coefficients. Next, a moderate mediation analysis (model 7) proposed by Hayes [31] was carried out. In this model, the simple mediation analysis is combined with the moderation analysis, allowing us to discover whether the mediating variable produces a differential effect in the different categories of the moderating variable. In this study, it was used to investigate whether pain catastrophizing (W) moderates the indirect effect of pain severity (X) on self-efficacy (Y) through pain avoidance (M) (Figure 1), controlling for the effect of the general index of symptoms, time elapsed since onset of symptoms, and time elapsed since FM Diagnosis. To include these variables in the models, it was first verified that they correlated with each other. Statistical significance was defined as a two-tailed *p*-value of <0.01. To test its statistical significance, the bootstrapping method with 5000 bootstrap samples was used to construct the 95% confidence intervals.

## 3. Results

### 3.1. Sample Characteristics

The participants’ mean age was 56.91 years (Standard Deviation, SD = 8.94). Only 15% of the women had completed higher education studies. The remaining participants reported having completed secondary (61%) or primary studies (24%). More than half of the women (53%) were married or in a stable relationship, 11% were single, and 36% of them were divorced or widowed. Most of the participants were housewives (76%). The mean score for the time elapsed since FM diagnosis was 12.14 years (SD = 8.45; range 1 to 46 years), and the mean score for time elapsed since onset of symptoms was 24.22 years (SD = 13.85; range 5 to 63 years).

### 3.2. Correlations

Table 1 shows the correlations among pain severity, pain catastrophizing, pain avoidance, self-efficacy, general symptom index, time elapsed since FM diagnosis, and time elapsed since onset of symptom. Self-efficacy was negatively correlated with pain severity, pain catastrophizing, pain avoidance, and general symptom index (*p* < 0.001). Pain avoidance was positively correlated with pain severity and pain catastrophizing (*p* < 0.001). Likewise, pain severity was positively correlated with pain catastrophizing, the general symptom index, time elapsed since FM diagnosis, and time elapsed since onset of symptoms (*p* < 0.05). The time elapsed since FM diagnosis correlated positively with the time elapsed since onset of symptoms (*p* < 0.05). 

### 3.3. Moderated Mediation Model

The moderated mediation analysis considers pain avoidance as a mediator when pain catastrophizing is used as a moderator in the relationship between pain severity and pain self-efficacy (see Table 2). The main effect of pain catastrophizing was significant, and the interaction between pain severity and pain catastrophizing was also significant (all *p* < 0.05). The general index of symptoms, time elapsed since FM diagnosis, and time elapsed since onset of symptoms were included as covariates. Thus, it is observed that the mediation of pain avoidance on the relationship between pain severity and self-efficacy was moderated by pain catastrophizing (Index = −0.027, SE = 0.017, 95% [Intervale Confidence IC= −0.068, −0.002]). This suggests that the effect of pain severity on self-efficacy through pain avoidance changes as a function of levels of pain catastrophizing.

**Figure 2:**
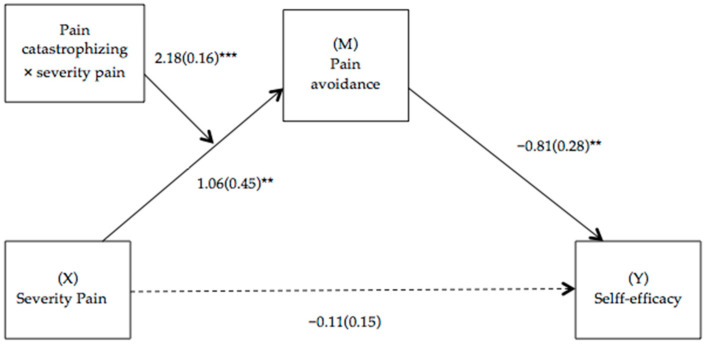
Path diagram illustrating the moderate mediation model. Notes: Values are non-standardized regression coefficients (SE in parentheses) and associated *p* values (*** *p* < 0.001, ** *p* < 0.01). Association in brackets = direct effect (controlling for indirect effects). Solid lines indicate important pathways and dashed lines indicate non-significant pathways.

To delimit the levels of statistical significance at which the effect of the independent variable (e.g., pain intensity) on the independent variable (e.g., pain avoidance) is specifically observed at the different values of the moderating variable (e.g., catastrophizing of pain), the pick-a-point selection method was used. It was plotted when levels of pain catastrophizing were one standard deviation below and one standard deviation above the mean. That is, it represents the simple effect of pain severity at two levels of pain catastrophizing (moderating variable). Figure 2 shows pain catastrophizing moderating the relationship between pain severity and pain avoidance when pain avoidance mediates the relationship between pain severity and self-efficacy. 

The proposed model explains 49% of the variance of pain self-efficacy. No multicollinearity problems were evident in the analyses (tolerance values above 0.010). A confidence interval based on 5000 resampled samples (bootstrap method) was considered to evaluate the conditional, indirect effects of pain intensity on pain self-efficacy through pain avoidance based on different levels of catastrophizing. The 95% CIs of this method were used to examine the indirect effects at three levels of catastrophizing (one standard deviation below, one standard deviation above the mean, and at the mean). The mediating effect of pain avoidance changed according to the level of pain catastrophizing and was weaker at 1 standard deviation below the mean (not-significant) (see Table 3). This indicates that fibromyalgia patients with severe pain were less likely to have self-efficacy through pain avoidance when they had higher pain catastrophizing (1 standard deviation above the mean). 

## 4. Discussion

Based on previous theories that point to pain avoidance as a maladaptive pattern in fibromyalgia patients, a pattern that, in turn, impedes the performance of activity and, consequently, the perception of competence derived from it [32], the hypothesis was raised that pain avoidance would mediate the relationship between pain severity and self-efficacy. Second, we expected that pain catastrophizing moderates the relationship between pain severity and pain avoidance according to research that suggests that catastrophic beliefs guide much attention toward pain, thus leading to greater disability [33]. Our results supported both hypotheses, providing evidence in favor of the affective-motivational models [34,35], in which catastrophizing and pain avoidance are pointed out as risk factors associated with fear of movement. However, certain positive psychological variables (e.g., self-efficacy) have not been investigated in-depth within these models.

The results found in the simple mediation model suggest that the impact of pain-avoidance pattern negatively influences self-efficacy beliefs. In line with Karsdorp’s and Vlaeyen’s suggestion [34], the mediating effect of the avoidance pattern, as an emotional regulation mechanism (in this case directed towards a hedonic goal such as pain avoidance), is confirmed. However, in line with the previous literature, this pattern is maladaptive in the long term, increasing functional limitation through activity avoidance [36,37,38]. The model tested in the present study additionally points to the negative effects of pain avoidance on the perception of self-efficacy, the latter being a fundamental resource in the symptomatology and quality of life of fibromyalgia patients [8,9,12]. 

In this context, self-efficacy is seen as one of the best health outcomes from goal-preference models in FM patients [10,36] due to it promoting adherence to physical exercise as one of the most effective multicomponent treatments in FM [4]. Thus, our results support the previous research by pointing out that patients with low levels of pain are predisposed to have cognitions of being able to effectively manage pain, despite barriers. Therefore, self-efficacy could act as a predictor of physical exercise in fibromyalgia as concluded by the previous literature [37,38]. In this line, our results may serve as a basis, with a special focus on the promotion of self-efficacy as a resource, for studies aimed at the effect of physical exercise, considering walking as the most recommended exercise in FM patients [39,40].

Additionally, our findings suggest that reducing self-efficacy through pain avoidance should additionally take into account the cognitions involved in the amplification of painful sensations (pain catastrophizing), as they may predispose FM patients to engage in behaviors to reduce pain intensity (pain avoidance). The moderated mediation model tested showed that patients experiencing high pain severity have a greater tendency to avoid pain, and this relationship is more intense when the levels of pain catastrophizing are higher [41]. Again, this is consistent with the fear-avoidance model [13,42]. For example, chronic-pain patients with perceived high levels of pain interpret pain as a reason that explains the problems of functionality, so an excessive fear of pain/injury appears to lead to the avoidance of physical activity. Furthermore, lack of activity leads to exacerbation of pain and disability [32]. This idea is also supported by affective-motivational models and their empirical findings which point to catastrophism as the factor responsible for the preference for pain control goals through inactivity [34,35]. In this line, the conditional effect was only significant for high pain catastrophizing, and our moderated mediation model was inconclusive for patients scoring low or medium on the pain catastrophizing scale.

### 4.1. Limitations

Firstly, it is necessary to be cautious when generalizing the results of our model as a cross-sectional design was used in this research. Secondly, caution must be taken when interpreting mediation models such as the one proposed in this work since causal relationships cannot be established, but attempts should rather be made to approximate the nature of fibromyalgia patients’ behavior in relation to pain self-efficacy from the psychological variables of interest. Therefore, future research with longitudinal designs would be necessary to determine if pain severity is maintained in the face of catastrophizing pain and, therefore, conditions the self-efficacy of patients to adhere to physical activity.

Third, the assessment measures used (e.g., self-report measures) are sensitive to various sources of error. Some of them are social desirability responding and recall bias. To mitigate these risks, we included the use of measures with good psychometric properties, which all demonstrated excellent reliability in our sample. Despite having a large sample that provided good statistical power, it was a convenience sample selected from several fibromyalgia associations. Therefore, only women participated. However, between 5 and 10% of fibromyalgia patients are men [43,44], so we could not generalize our results. 

### 4.2. Clinical Implications and Future Research

These limitations could be overcome in future research. Specifically, longitudinal designs could be used to replicate these findings in large samples on fibromyalgia, but, more importantly, future research can apply these findings in clinical practice with patients with chronic pain.

Our results point towards interventions aimed at attenuating catastrophic cognitions about activity and pain as a strategy to reduce avoidant behavior and generate self-efficacy [45,46]. According to different guidelines on multidisciplinary treatments in fibromyalgia [47,48], psychological treatment focused on ruminations and catastrophic beliefs is recommended so that patients can resolve the internal conflict established by the different health goals (pain control vs. physical activity). Different techniques have shown their usefulness for this objective, such as motivational interviewing [49,50]. Motivation and commitment are the two central components of motivational interviewing, and fear of movement and catastrophizing are precisely the main risk factors when initiating or maintaining activity patterns instead of avoidance patterns. Therefore, our findings support the need to adapt these types of techniques focusing on cognitions to improve patients’ self-efficacy and, therefore, their quality of life. Consequently, patients are reinforced to obtain a realistic perception of their symptomatology so that it does not limit their daily life. This study reinforces the rationale for addressing pain catastrophizing clinically. 

## 5. Conclusions

Taking into account the limitations mentioned above, this study suggests that pain avoidance mediates the relationship between pain severity and self-efficacy, and this is maximized in FM patients with higher levels of pain catastrophizing. These patients present with maladaptive beliefs about pain, including constant worries about whether it will go away, about the ability to bear it, about the future interference of pain in life, and, in short, about not being able to forget the pain. This suggests that pain catastrophizing is a self-regulatory strategy that could ultimately be counterproductive. Longitudinal studies are needed to verify the findings found here.

## Figures and Tables

**Table 1:** Descriptive statistics of psychosocial characteristics (n = 264).

Psychosocial Characteristics	Mean (SD)	Sample Range	2.	3.	4.	5.	6.	7.
1. Pain severity	7.40 (1.80)	1–10	0.309 **	0.268 *	−0.234 *	0.330 **	0.161 *	0.234 **
2. Pain catastrophizing	29.73 (11.24)	6–52		0.220 *	−0.481 **	0.360 **	0.057	−0.029
3. Pain avoidance	6.76 (2.78)	0–12			−0.270 **	0.065	0.284	0.081
4. Self-efficacy	14.31 (9.02)	0–35				−0.345 **	−0.051	−0.020
5. General index of symptoms	9.55 (1.77)	3–12					−0.043	0.106
6. Diagnosis of FM	12.32 (2.82)	1–46						0.541 **
7. Duration of FM	24.22 (13.85)	5–63						

Abbreviations: SD (standard deviation), FM = Fibromyalgia, ** *p* < 0.01, * *p* < 0.05.

**Table 2:** Moderate mediation analysis assuming pain catastrophizing as moderator.

VD: Pain Avoidance	*R* ^2^	*F*	*p*	Beta	*t*	*p*
Model summary	0.38	2.71	0.010			
VI: Pain severity				−0.92	−2.14	0.034
W: Pain catastrophizing				−0.17	−1.90	0.05
Pain catastrophizing × Severity pain				0.02	2.36	0.001
General index of symptoms (covariate)				0.08	0.54	0.583
Time since FM diagnosis (covariate)				0.02	0.38	0.702
Time since onset of symptoms (covariate)				0.04	0.32	0.743
**VD: Self-efficacy**	***R* ^2^**	***F***	***p***	**Beta**	***t***	***p***
Model summary	0.49	6.21	<0.001			
VI: Pain severity				−0.59	0.51	0.249
M: Pain avoidance				−0.91	0.30	0.004
General index of symptoms (covariate)				0.08	0.54	0.583
Time since FM diagnosis (covariate)				0.02	0.38	0.702
Time since onset of symptoms (covariate)				0.04	0.32	0.743

**Table 3:** Indirect conditional effect at specific levels of the moderator (pain catastrophizing) when treating pain avoidance as a mediator.

Pain Catastrophizing	Beta	SE	LL 95% CI	UL 95% CI
1SD below the mean	0.35	0.27	−0.06	1.01
Mean	0.05	0.17	−0.24	0.41
1SD above the mean	−0.24	0.26	−0.77	−0.04

Notes: SE = standard error; LL 95% CI = lower level of the 95% confidence interval; UL 95% CI = upper level of the 95% confidence interval.

## Data Availability

The data presented in this study are available on request from the corresponding author. The data are not publicly available due to privacy restrictions.
